# The Potential of MET Immunoreactivity for Prediction of Lymph Node Metastasis in Early Oral Tongue Squamous Cell Carcinoma

**DOI:** 10.3389/fonc.2021.638048

**Published:** 2021-04-29

**Authors:** Maria J. De Herdt, Berdine van der Steen, Quincy M. van der Toom, Yassine Aaboubout, Stefan M. Willems, Marjan H. Wieringa, Robert J. Baatenburg de Jong, Leendert H. J. Looijenga, Senada Koljenović, Jose A. Hardillo

**Affiliations:** ^1^ Department of Otorhinolaryngology and Head and Neck Surgery, Erasmus MC, University Medical Center Rotterdam, Cancer Institute, Rotterdam, Netherlands; ^2^ Department of Pathology and Medical Biology, Erasmus MC, University Medical Center Rotterdam, Cancer Institute, Rotterdam, Netherlands; ^3^ Department of Pathology, University Medical Center Groningen, Groningen, Netherlands; ^4^ Department of Education, Office of Science, Elisabeth TweeSteden, Ziekenhuis, Tilburg, Netherlands; ^5^ Princess Maxima Center for Pediatric Oncology, Utrecht, Netherlands

**Keywords:** MET, occult lymph node metastasis, depth of invasion, oral tongue squamous cell carcinoma, elective neck dissection

## Abstract

**Objective:**

MET positivity is independently associated with survival in oral squamous cell carcinoma (OSCC). Since MET is a known orchestrator of invasive tumor growth, we investigated its association with LNM in early oral tongue squamous cell carcinoma (OTSCC). As it is recommended by the NCCN to use tumor depth of invasion (DOI) in making decisions on elective neck dissection (END), the results obtained for MET positivity were aligned with those for DOI > 4 mm. The cutoff value used in our institution.

**Methods:**

Tumor samples from patients who underwent primary tumor resection and neck dissection between 1995 and 2013, were collected from the archives of the Leiden and Erasmus University Medical Center. Immunohistochemistry with D1C2 was performed to identify MET negative (< 10% uniform positivity) and MET positive (≥ 10% uniform positivity) cancers. ROC curve analysis and the Chi-squared test were used to investigate the association of MET positivity with LNM (pN+ and occult). Binary logistic regression was used to investigate the association of MET positivity with LNM.

**Results:**

Forty-five (44.1%) of the 102 cancers were MET positive. Ninety were cN0 of which 20 were pN+ (occult metastasis). The remaining 12 cancers were cN+, of which 10 were proven pN+ and 2 were pN0. MET positivity was associated with LNM with a positive predictive value (PPV) of 44.4% and a negative predictive value (NPV) of 82.5% for pN+. For the occult group, the PPV was 36.8% and the NPV was 88.5%. Regression analysis showed that MET positivity is associated with pN+ and occult LNM (*p*-value < 0.05).

**Conclusion:**

MET positivity is significantly associated with LNM in early OTSCC, outperforming DOI. The added value of MET positivity could be in the preoperative setting when END is being considered during the initial surgery. For cases with DOI ≤ 4 mm, MET positivity could aid in the clinical decision whether regular follow-up, watchful waiting, or END is more appropriate. Realizing that these preliminary results need to be independently validated in a larger patient cohort, we believe that MET positivity could be of added value in the decision making on END in early OTSCC.

## Introduction

Approximately one third of head and neck squamous cell carcinoma (HNSCC) originate in the oral cavity (OSCC) (1). For patients diagnosed with OSCC with clinically positive cervical lymph nodes, primary tumor resection with neck dissection is indicated. On the other hand, elective neck dissection (END) is recommended if the risk of occult lymph node metastasis (LNM) is 20% (2). To date, tumor depth of invasion (DOI) is an established predictor for occult LNM and is recommended by the NCCN in making decisions on END (1).

Depth of invasion with a cut-off value (> 4 mm) is a strong predictor for occult LNM, this cutoff value is therefore used within the Erasmus MC in making decisions on END (1, 3, 4). The DOI however is determined during the final pathological evaluation, days after the excision of the primary tumor (5). Therefore, cancers with DOI of > 4 mm, would necessitate a second stage END resulting in additional morbidity for the patient, inefficient use of resources, time, and extra costs. Another downside of DOI is that it has been used interchangeably with tumor thickness, another predictor of LNM (6–9). This problem has been addressed in the 8^th^ edition of the AJCC that provides a clear definition for DOI (5).

In some centers, sentinel lymph node biopsy (SLNB) is being performed to rule out the presence of occult LNM. With detection rates of 95% (10–12), 0.93 sensitivity and NPV of 0.88 to 1 (11–15), SLNB is a reliable method to detect occult LNM during surgery. However, the success rate of SLNB depends on the experience and technical expertise of the team performing the procedure making the implementation of SLNB in routine patient care difficult (1).

It is clear, that there still is a need for reliable preoperative predictors of occult LNM that can further improve the decision making process on END. Ideally, such predictors should be easily incorporated in a routine diagnostic setting.

A target of interest is the receptor tyrosine kinase MET (16). Using a novel scoring system, it was shown that MET positivity is associated with poor overall survival (OS) and disease-free survival (DFS) in OSCC (17). Amongst its pleiotropic functions as an oncogene, *MET* orchestrates the program of invasive growth (16, 18, 19). As MET facilitates the dissemination of cancers cells, it is an interesting target for the prediction of LNM.

This study investigated whether MET positivity is associated with LNM (pN+ and occult) in early oral tongue SCC (OTSCC). This association was compared with DOI > 4 mm, a known predictor of occult LNM. We further, hypothesized on the potential added value of MET positivity in the clinical decision-making on END.

## Materials and Methods

### Ethics Statement

Human tissues and patient data were used according to “The Code of Conduct for Responsible Use” and “The Code of Conduct for Health Research” as stated by the Federation of Dutch Medical Scientific Societies (20). Furthermore, The Erasmus MC Medical Ethics Committee approved the research protocol (MEC-2016-751).

### Study Design, Patient, and Tumor Characteristics

Inclusion criteria were patients with early OTSCC (pT1-2) treated primary with surgery with a neck dissection, at the Leiden University Medical Center (LUMC, between 1995 and 2010) and the Erasmus MC Cancer Institute (Erasmus MC, between 2006 and 2013).

All patient and tumor characteristics, except the DOI, were retrieved from the patient files including: gender, age, tumor diameter (cm), TNM (21), extranodal extension (21), and margin status (21, 22). The DOI was measured according to the guidelines of the 8th edition of the AJCC (5). HE sections—made for diagnostic assessment—were retrieved from the archives and scanned using the NanoZoomer 2.0-HT slide scanner (Hamamatsu Photonics, Hamamatsu, Japan). Other tumor characteristics (i.e. degree of differentiation, pattern of invasion, lymphovascular invasion, perineural invasion, and data on postoperative radiotherapy) were not further recorded or analyzed in this study.

Follow-up data in respect to OS and DFS were also recorded.

A previous history of head and neck cancer was a reason for exclusion.

### Tissues, Antibody, and Immunohistochemistry

Formalin-fixed paraffin-embedded tissue blocks representative of the included cancers were retrieved from the tissue banks of the departments of pathology from both medical centers. Using a microtome, 3 µm thick whole tissue sections were cut for immunohistochemical analyses.

D1C2 (Cell Signaling Technology^®^; Danvers, MA, USA) was used to detect C-terminal MET immunoreactivity according to the method described in our previous publications (17, 23).

**Figure 1:**
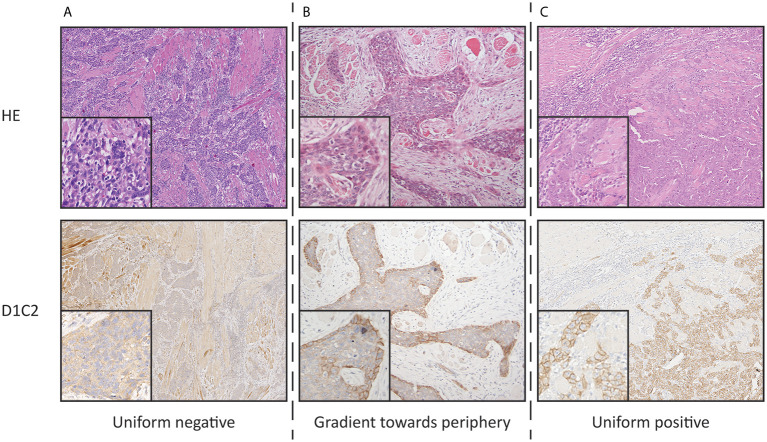
Photographs representing the defined staining patterns observed using D1C2 and corresponding HE section (10× and 20× objective). **(A)** Uniform negative **(B)** Gradient toward periphery. **(C)** Uniform positive. The gradient toward center staining pattern was not observed.

Membranous immunoreactivities obtained using D1C2 were characterized by assessing for four staining patterns: uniform negative, gradient toward the periphery, uniform positive, and gradient toward the center according to the scoring system described in our previous publication (**Figure 1**) (17).

### Association of MET Positivity and DOI With LNM

Analogous to the known association of ≥ 10% of the D1C2 uniform positive staining pattern with OS and DFS, it was assessed if ≥ 10% D1C2 uniform positivity (further on referred to as MET positivity) is associated with histopathologically proven LNM (cN0/pN+ and cN+/pN+) and occult LNM (cN0/pN+) using receiver operating characteristic (ROC) curve analysis (17).

The DOI cutoff value > 4 mm used in the Erasmus MC as an indication for END, was investigated by assessing the association between DOI > 4 mm and proven as well as occult LNM using ROC curve analysis (1).

## Statistical Analyses

The Chi-squared test, non-parametric Fisher’s exact test and independent-Samples T-test were used to compare the patient and tumor characteristics of the LUMC and Erasmus MC cohorts.

To calculate the proportion of MET positive cancers within the whole pN+ group (occult and overt LNM), the Chi-squared test was used. The same was done for DOI > 4 mm.

To calculate the proportion of MET positive cancers within the occult LNM group (cN0/pN+), the Chi-squared test was used. The same was done for DOI > 4 mm.

Binary logistic regression was performed to investigate whether MET positivity and/or DOI have a—joint—effect on LNM.

Calculations were performed with SPSS Statistics (version 25; IBM; Armonk, NY, USA). Unless otherwise mentioned, statistical significance was set at *p*-value <0.05.

## Results

### Comparison of the LUMC and Erasmus MC Patient and Tumor Characteristics

The 102 patients included in this study were treated for primary pT1-2 OTSCC with surgery and—if indicated—postoperative radiotherapy in the LUMC or Erasmus MC across different periods. Twenty-five (24.5%) patients were treated in the LUMC and 77 (75.5%) in the Erasmus MC. Comparison of the patient and tumor characteristics as well as MET positivity shows that there are no differences between the two centers (**Supplementary Table 1**). OS and DFS were also similar (results not shown).

### Association of MET Positivity and DOI With LNM in D1C2 Positive Cancers

Hundred and two (102) patients were treated with tumor resection and a neck dissection. Thirty (29.4%) were pN+, of which 20 (66.7%) were cN0 and therefore occult metastases (**Table 1**).

**Table 1:** Presentation of pathological lymph node groups (pN) with respect to clinical lymph node groups (cN).

		cN		Total
		N0	N+	
pN	N0	70	2	72
N+		20	10	30
Total		90	12	102

**Figure 2:**
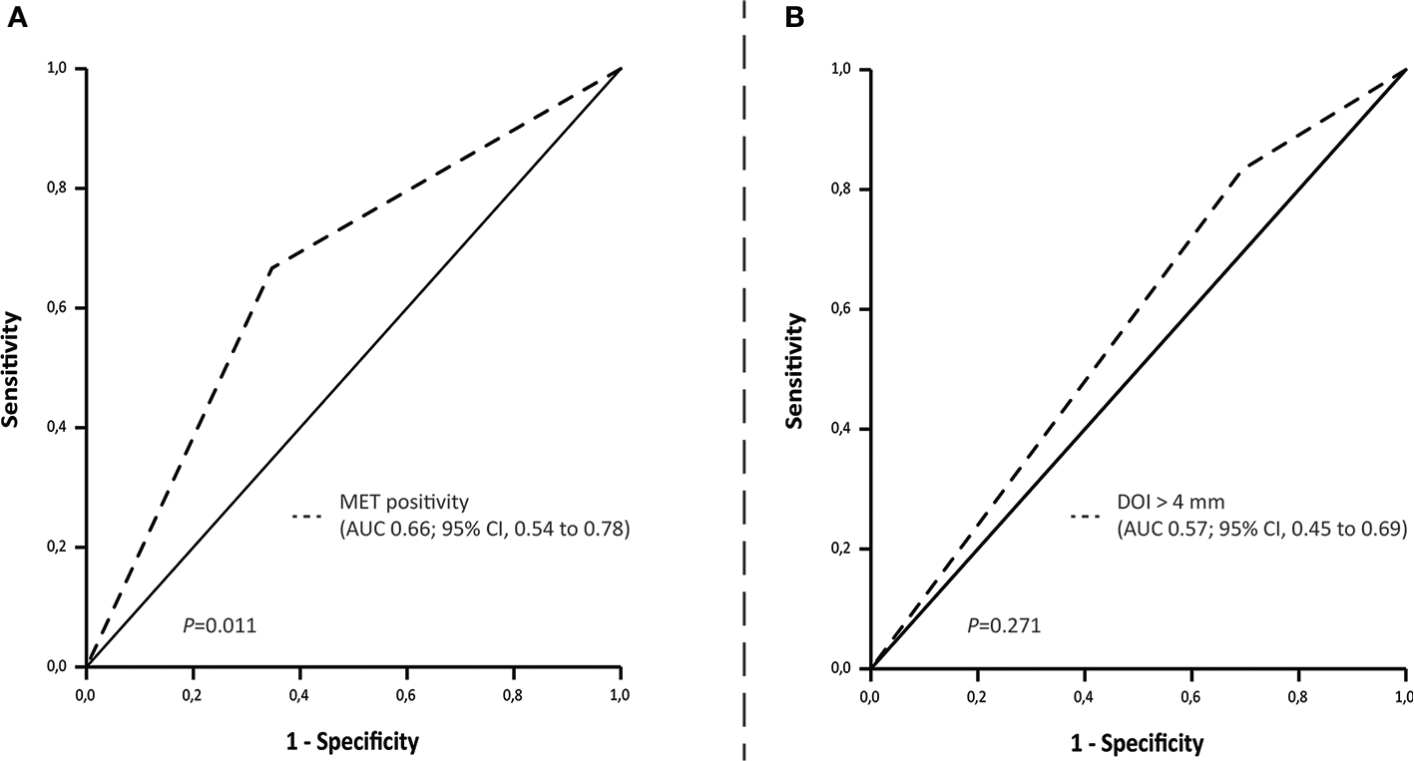
ROC curve indicating the area under the curve for **(A)** MET positivity and **(B)** Depth of invasion set at > 4 mm and LNM (cN0 and cN+/pN+).

ROC curve analyses showed that MET positivity is associated with pN+ and occult LNM and that DOI > 4 mm has a higher sensitivity for pN+ and occult LNM compared to MET positivity (**Figure 2**, **Supplementary Figure 1**).

Forty-five (44.1%) of the included cancers were positive for MET (**Table 2**). Seventy-five cancers (73.5%) had a DOI > 4 mm (**Table 3**). The positive predictive value (PPV) for MET positivity was 44.4%, for DOI > 4 mm 33.3% (**Table 4**). The NPV for MET positivity was 82.5%, for DOI > 4 mm 81.5% (**Table 4**). Within the cN0 group (n=90), 38 cancers were positive for MET (42.2%) (**Table 2**) and 52 had a DOI > 4 mm (57.8%) (**Table 3**). For this cN0 group, the PPV for MET positivity was 36.8%, for DOI > 4 mm 25.8%; the NPV for MET positivity was 88.5% and for DOI > 4 mm 87.5% (**Table 4**).

**Table 2:** Cross-tabulations showing the relationship between MET positivity and pN status in all cancers (n=102) and in cN0 cancers (n=90).

MET positivity	All patients (n = 102)			cN0 patient (n = 90)		
	pN+	pN0	Row total	pN+	pN0	Row total
Yes	20 (66.7%)	25 (34.7%)	45 (44.1%)	14 (70.0%)	24 (34.3%)	38 (42.2%)
No	10 (33.3%)	47 (65.3%)	57 (55.9%)	6 (30.0%)	46 (65.7%)	52 (57.8%)
Column total	30 (100%)	72 (100%)	102 (100%)	20 (100%)	70 (100%)	90 (100%)
Significance	*p*-value = 0.003			*p*-value = 0.004		

**Table 3:** Cross-tabulations showing the relationship between DOI and pN status in all cancers (n=102) and in cN0 cancers (n=90).

DOI	All patients (n=102)			cN0 patient (n=90)		
	pN+	pN0	Row total	pN+	pN0	Row total
> 4 mm	25 (83.3%)	50 (69.4%)	75 (73.5%)	17 (85.0%)	49 (70.0%)	66 (73.3%)
≤ 4 mm	5 (16.7%)	22 (30.6%)	27 (26.5%)	3 (15.0%)	21 (30.0%)	24 (26.7%)
Column total	30 (100%)	72 (100%)	102 (100%)	20 (100%)	70 (100%)	90 (100%)
Significance	*p*-value = 0.147			*p*-value = 0.181		

**Table 4:** Sensitivity, specificity, PPV, and NPV and accuracy of MET positivity and DOI > 4 mm with respect to pN+ for all cancers (n=102) and cN0 cancers (n=90).

	All patients (n = 102)					cN0 patients (n = 90)				
	Sens. (%)	Spec. (%)	PPV (%)	NPV (%)	Acc. (%)	Sens. (%)	Spec. (%)	PPV (%)	NPV (%)	Acc. (%)
MET positivity	66.7	65.3	44.4	82.5	65.7	70.0	65.7	36.8	88.5	66.7
DOI > 4 mm	83.3	30.6	33.3	81.5	46.1	85.0	30.0	25.8	87.5	42.2

### MET Positivity and DOI as Predictors for (pN+ and Occult) LNM

Univariable binary logistic regression showed that MET positivity is associated with pN+ status in general (OR = 3.76; 95% CI: 1.53–9.26, *p*-value < 0.05; **Supplementary Table 2**) and for occult LNM (OR = 4.28; 95% CI: 1.45–12.65, *p*-value < 0.05; **Supplementary Table 2**). DOI > 4 mm shows an OR of 2.20 for pN+ (95% CI: 0.745–6.50, *p*-value = 0.15; **Supplementary Table 3**) and 2.43 for occult LNM (95% CI: 0.64–9.18, *p*-value = 0.19; **Supplementary Table 3**). Multivariable analysis showed that MET positivity is independently associated with pN+ in general and occult LNM when corrected for DOI > 4 mm (**Table 5**).

**Table 5:** Multivariable binary logistic regression model investigating the independent effect of MET positivity and DOI > 4 mm on pN+ for all cancers (n = 102) and cN0 cancers (n = 90).

Variable	All patients (n = 102)			cN0 patient (n = 90)		
	Odds ratio	95% CI	*p*-value	Odds ratio	95% CI	*p*-value
MET positivity	3.66	1.47–9.06	0.005	4.28	1.45–12.65	0.009
DOI > 4 mm	2.05	0.67–6.30	0.209	2.16	0.55–8.53	0.274
Constant	0.12		0.000	0.073		0.000
Significance	*p*-value = 0.005			*p*-value = 0.009		

A 2 × 2 table for pN+ cancers (n=30), depicting the number of cancers either negative (< 10% uniform positivity) or positive for MET versus DOI ≤ or > 4 mm, illustrates that there were 2 (6.67%) MET negative cancers with LNM in the group DOI ≤ 4 mm. Three (10.0%) cancers with LNM were MET positive and had a DOI ≤ 4 mm. Eight (26.7%) cancers with LNM were MET negative and had a DOI > 4 mm. Seventeen (56.7%) cancers with LNM were MET positive and had a DOI > 4 mm (**Table 6**). A similar 2 × 2 table for cases with occult LNM (n=20) shows 1 (5.00%) MET negative cancer with occult LNM with DOI ≤ 4 mm. Two (10.0%) cancers with occult LNM were MET positive and had a DOI ≤ 4 mm. Five (25.0%) cancers with occult LNM were MET negative and had a DOI > 4 mm. Twelve (60.0%) cancers with occult LNM were MET positive and had a DOI > 4 mm (**Table 6**). These numbers illustrated the potential additive value of MET positivity to DOI > 4 mm to assess the presence of LNM (pN+ and occult).

**Table 6:** 2 × 2 tables showing the relationship between MET positivity and DOI status in cancers with pN+ (n=30) and cancers with occult LNM (n=20).

	Cancers with pN+ (n=30)		Cancers with occult LNM (n=20)	
	DOI ≤ 4 mm	DOI > 4 mm	DOI ≤ 4 mm	DOI > 4 mm
MET negative	2 (6.67%)	8 (26.7%)	1 (5.00%)	5 (25.0%)
MET positive	3 (10.0%)	17 (56.7%)	2 (10.0%)	12 (60.0%)

## Discussion

For patients with early OSCC, END is generally recommended when the chance of occult lymph node metastasis is more than 20% (2, 4, 24–26). DOI is one of the most reliable parameters to predict occult LNM and guide clinical decision making on END. At our center DOI cut-off value > 4 mm is used. DOI is usually determined days after initial surgery based on the final histopathological assessment. As such, END is often performed during second surgery when DOI is > 4 mm. There is a need for reliable measurement of DOI before initial cancer surgery for example in biopsies. However, DOI measured on diagnostic biopsies is not reliable as sampling may not be representative of the entire primary cancer (24, 27). Assessment of DOI by preoperative MRI or intraoral ultrasonography is an alternative. However, preoperative MRI has been reported to be not accurate in tumors with DOI < 5 mm (28–33). Another alternative is measuring DOI during specimen-driven intraoperative assessment using frozen sections (34–36). This would enable an elective END during initial surgery in cN0 cases with DOI > 4 mm. Although promising, further validation and optimization is necessary to implement intraoperative assessment of DOI using frozen sections in a routine diagnostic setting. However, intraoperative assessment of DOI also has its limitations entailing patient uncertainty concerning the decision on END prior to surgery and the unnecessary prescheduled OR time for all potential ENDs leading to inefficiency and additional costs. Moreover, the direct communication that is necessary between surgeon and pathologist is not always possible (24, 36).

The current study shows that MET positivity is univariably associated with LNM in OTSCC. This result was expected given the established association of MET positivity with OS and DFS in OSCC (17, 23). The result also concurs with the fact that wildtype MET activity is known to increase cell death, invasion and distant metastasis (18).

Receptor tyrosine kinase MET is a known orchestrator of invasive growth (16, 19, 37, 38). As LNM is one of the major determinants of patient outcome in HNSCC, we are not the first to investigate the association between MET expression and LNM. It has been shown that expression levels of MET are high in cancer tissues and in corresponding affected lymph nodes (39–41). Additionally, it was shown that the *MET* gene product is more sensitive in the detection of occult LNM compared to cytokeratins in OSCC (42).

The PPV of MET on occult LNM was 36.8%, which meets the recommendation of performing an END if the risk of occult LNM is 20% (NPV 80%) (2). In this study, we also showed that MET positivity has a NPV of 88.5% for occult LNM. Depth of invasion > 4 mm is a known predictor for occult LNM (4), in accordance with our results (PPV 25.8%). The established PPVs of MET positivity and DOI > 4mm showed that MET outperforms DOI in predicting occult LNM. Multivariable analysis showed that only MET positivity is independently associated with LNM, pN+ and occult (*p*-value < 0.05). Although DOI > 4 mm did not significantly contribute to the multivariable model, it showed a strong relation with pN+ LNM (OR=2.05, *p*-value = 0.209) and occult LNM (OR=2.16, *p*-value = 0.274). The lack of significance of DOI > 4 mm can probably be explained by the low number of cases with DOI ≤ 4mm. These results suggest that MET positivity might also be of value in predicting the presence or absence of occult LNM in the preoperative setting, and besides DOI, in the postoperative setting (after removal of the primary tumor) in early OTSCC.

We foresee that performing routine immunohistochemistry using the D1C2 antibody against the C-terminus of MET on biopsies, could be of great value in deciding when and when not to perform an END during the initial OTSCC surgery. This would improve logistics, cost-effectiveness, and would reduce patient morbidity caused by two separate surgeries. This study is performed on whole tissue sections, therefore efforts have to be made to design a future study to extrapolate these results to biopsies. To anticipate for cancer heterogeneity we expect that (preoperative) biopsies will need to be taken from the center and periphery of the cancer (17).

The fact that there were less occult LNMs for cancers negative for MET and with DOI ≤ 4 mm compared to cancers positive for MET and with DOI ≤ 4 mm, illustrates that MET positivity could be of added value to DOI ≤ 4 mm for the clinical decision on the treatment of the cN0 neck i.e., whether regular follow-up, watchful waiting, or END is more appropriate. We can imagine that patients with MET positive cancers and DOI ≤ 4 mm could have more stringent follow-up than patients in the same DOI group but with MET negative cancers. We realize that this statement is based on very low numbers and independent validation is necessary.

Taking into account that the obtained results are preliminary and need to be independently validated in a larger and more recent patient cohort incorporating both patients that underwent END and watchful waiting and containing detailed registration of relevant histopathological parameters, we believe that MET positivity could be of added value in pre- and postoperative (after primary tumor resection) decision making on END in early OTSCC.

## Data Availability Statement

The raw data supporting the conclusions of this article will be made available by the authors, without undue reservation.

## Ethics Statement

Human tissues and patient data were used according to “The Code of Conduct for Responsible Use” and “The Code of Conduct for Health Research” as stated by the Federation of Dutch Medical Scientific Societies. Furthermore, The Erasmus MC Medical Ethics Committee approved the research protocol (MEC-2016-751).

## Author Contributions

MD: conceptualization, methodology, software, formal analysis, data curation, writing—original draft, and visualization. BV: investigation, data curation, writing—review and editing, and visualization. QV: data curation and writing—review and editing. YA: data curation and writing—review and editing. SW: writing—review and editing. MW: methodology and writing—review and editing. RB: writing—review and editing. LL: writing—review and editing. SK: conceptualization, methodology, writing—review and editing, and supervision. JH: conceptualization, methodology, writing—review and editing, and supervision. All authors contributed to the article and approved the submitted version.

## Conflict of Interest

The authors declare that the research was conducted in the absence of any commercial or financial relationships that could be construed as a potential conflict of interest.
