# Supplementary material for: The Potential of MET Immunoreactivity for Prediction of Lymph Node Metastasis in Early Oral Tongue Squamous Cell Carcinoma
Source: Front Oncol. 2021 Apr 29;11:638048. doi: 10.3389/fonc.2021.638048 (PMC8117234; doi:10.3389/fonc.2021.638048)
Supplement: Supplementary file 1 [file DataSheet_1.docx]

# Supplementary tables & figures

Supplementary table 1: comparison of the patient & tumor characteristics and MET positivity between the early OTSCC included from the LUMC and Erasmus MC (n=102).

|  | | Leiden cohort (n=25) | | Rotterdam cohort (n=77) | | *p*-value |
| --- | --- | --- | --- | --- | --- | --- |
|  |  | Number | Percentage | Number | Percentage |  |
| Gender | | | | | | |
|  | Male | 14 | 56.0 | 46 | 59.7 |  |
|  | Female | 11 | 44.0 | 31 | 40.3 | 0.741 |
| Age at diagnosis (years) | | | | | | |
|  | Mean (range) | 57.0 (38.0 – 78.0) | | 60.5 (24.0 – 90.0) | | 0.283 |
| Tumor diameter (cm) | | | | | | |
|  | Mean (range) | 1.94 (0.700 – 3.10) | | 1.81 (0.400 – 4.00) | | 0.458 |
| pT (8^th^ edition of the AJCC) | | | | | | |
|  | T1 | 4 | 16.0 | 27 | 35.1 |  |
|  | T2 | 21 | 84.0 | 50 | 64.9 | 0.072 |
| pN | | | | | | |
|  | N0 | 16 | 64.0 | 56 | 72.7 |  |
|  | N≥1 | 9 | 36.0 | 21 | 27.3 | 0.405 |
| Extranodal extension^*^ | | | | | | |
|  | No | 22 | 88.0 | 72 | 93.5 |  |
|  | Yes | 3 | 12.0 | 5 | 6.5 | 0.401 |
| Pathological cancer stage^**^ (8^th^ edition of the AJCC) | | | | | | |
|  | I – II | 16 | 64.0 | 57 | 74.0 |  |
|  | III – IV | 9 | 36.0 | 20 | 26.0 | 0.334 |
| Resection margins | | | | | | |
|  | Clear and close | 21 | 84.0 | 60 | 77.9 |  |
|  | Positive | 4 | 16.0 | 17 | 22.1 | 0.514 |

| MET positive | | | | | | |
| --- | --- | --- | --- | --- | --- | --- |
|  | < 10% D1C2 uniform positivity | 10 | 40.0 | 47 | 61.0 |  |
|  | ≥ 10% D1C2 uniform positivity | 15 | 60.0 | 30 | 39.0 | 0.066 |
| DOI > 4 mm | | | | | | |
|  | ≤ 4 mm | 5 | 20.0 | 22 | 28.6 |  |
|  | > 4 mm | 20 | 80.0 | 55 | 71.4 | 0.399 |

^*^ Here the Fisher’s Exact Test was used as 1 cell has an expected count of less than 5.

^**^ All included patients are assessed as pM=0.

Supplementary table 2: univariable binary logistic regression model investigating the effect of MET positivity on pN+ for all cancers (n=102) and cN0 cancers (n=90).

| Variable | All patients (n=102) | | | cN0 patient (n=90) | | |
| --- | --- | --- | --- | --- | --- | --- |
|  | Odds ratio | 95% CI | *p*-value | Odds ratio | 95% CI | *p*-value |
| MET positivity | 3.76 | 1.53 – 9.26 | 0.004 | 4.47 | 1.53 – 13.1 | 0.006 |
| Constant | 0.21 |  | 0.000 | 0.13 |  | 0.000 |
| Significance | *p*-value = 0.003 | | | *p*-value = 0.004 | | |

Supplementary table 3: univariable binary logistic regression model investigating the effect of DOI > 4 mm on pN+ for all cancers (n=102) and cN0 cancers (n=90).

| Variable | All patients (n=102) | | | cN0 patient (n=90) | | |
| --- | --- | --- | --- | --- | --- | --- |
|  | Odds ratio | 95% CI | *p*-value | Odds ratio | 95% CI | *p*-value |
| DOI > 4 mm | 2.20 | 0.75 – 6.50 | 0.154 | 2.43 | 0.64 – 9.18 | 0.191 |
| Constant | 0.23 |  | 0.003 | 0.14 |  | 0.002 |
| Significance | *p*-value = 0.135 | | | *p*-value = 0.162 | | |


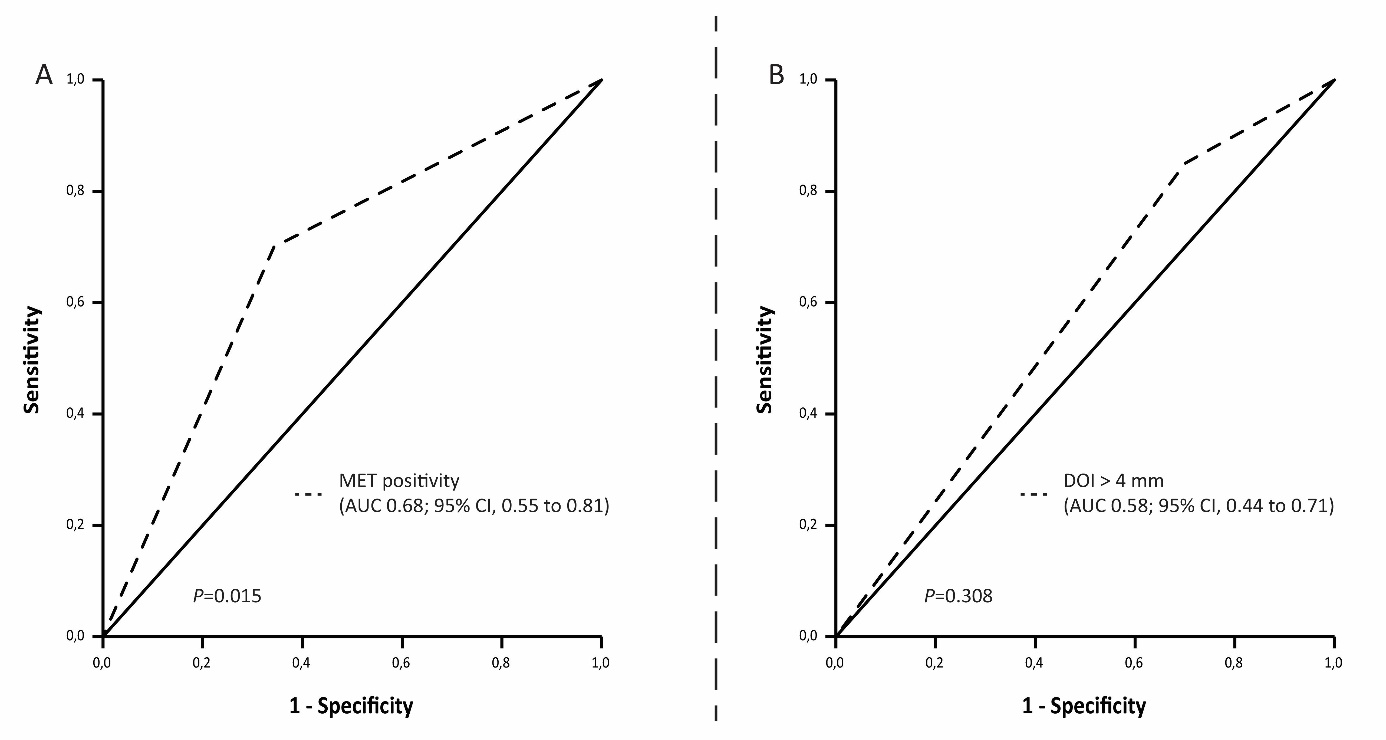


Supplementary figure 1: ROC curve indicating the area under the curve for A. MET positivity and B. Depth of invasion set at > 4 mm and occult LNM (cN0/pN+).
